# In a single-blind, matched group design: branched-chain amino acid supplementation and resistance training maintains lean body mass during a caloric restricted diet

**DOI:** 10.1186/s12970-015-0112-9

**Published:** 2016-01-05

**Authors:** Wesley David Dudgeon, Elizabeth Page Kelley, Timothy Paul Scheett

**Affiliations:** Department of Health and Human Performance, College of Charleston, 24 George Street, Charleston, SC 29424 USA

**Keywords:** Cut diet, Fat mass, Lean mass

## Abstract

**Background:**

Athletes and active adults many times have the goal of improving/maintaining fitness while losing weight and this is best achieved by caloric restriction in combination with exercise. However, this poses a risk for lean tissue loss, which can limit performance. Thus, the purpose of this study was to determine the effectiveness of a branched-chain amino acid (BCAA) supplement, in conjunction with heavy resistance training and a carbohydrate caloric-restricted “cut diet” on body composition and muscle fitness.

**Methods:**

Seventeen resistance-trained males (21–28 years of age) were randomized to a BCAA group (*n* = 9) or a carbohydrate (CHO) group (*n* = 8) who both received their respective supplement during the 8 weeks of a prescribed body building style resistance training protocol. Subjects were prescribed a hypocaloric diet (based upon pre-intervention analysis) that was to be followed during the study.

**Results:**

The BCAA group lost fat mass (−0.05 ± 0.08 kg;*p* < .05) and maintained lean mass, while the CHO group lost lean mass (−0.90 ± 0.06 kg; *p* < .05) and body mass (−2.3 ± 0.7 kg; *p* < .05). Both groups increased 1RM squat, but the increase in the BCAA group (15.1 ± 2.2 kg; p < .05)was greater (*P* < 0.05) than the CHO group. The BCAA group increased 1RM bench press (7.1 ± 1.6 kg; *P* < 0.05), while the CHO group decreased strength (−3.7 ± 2.3 kg; *P* < 0.05). The only change in muscular endurance was an increase in repetitions to fatigue (5.3 ± 0.2; *p* < .05) in the CHO group.

**Conclusion:**

These results show that BCAA supplementation in trained individuals performing resistance training while on a hypocaloric diet can maintain lean mass and preserve skeletal muscle performance while losing fat mass.

## Background

The prevalence of age and lifestyle-induced obesity among adults is increasing rapidly [1]. Thus, many adults engage in intentional weight loss, primarily via reductions in fat mass, to achieve aesthetic, performance, and/or health goals, including reduced risk for chronic disease and disability [2]. Weight loss can be achieved via a reduction in calorie intake in conjunction with the initiation of physical activity [1]. The “cut diet” is a well-known dieting technique in which calorie and carbohydrate restriction reduces carbohydrate stores in the body and increases fat utilization as fuel, which in turn reduces fat mass.

Resistance training is a common training modality that elicits significant muscular and cardiometabolic benefits among both recreational and elite athletes [3]. Resistance training stimulates muscle metabolism for muscle growth and development [4]. When performed regularly, resistance training has been shown to increase strength, muscular endurance, skeletal muscle hypertrophy, as well as result in favorable changes in body composition, including decreases in body fat mass and increases in lean mass, all of which can improve health-related quality of life [4–6].

However, maintaining an energy deficient diet during a period of intense or unaccustomed resistance training may lead to significant losses in lean mass and decrease work output, thus hindering athletic performance, as well as increasing the risk for acute illness and training-related injury [5, 7, 8]. Muscle damage, characterized by increased muscle and whole-body protein turnover and amino acid oxidation during and following exercise, increases the athlete’s need for protein intake [9]. Therefore, it is important for athletes and recreationally active adults who engage in higher intensity or resistance training programs, as well as adults at risk for sarcopenia, to maintain a protein intake that can sustain lean body mass for functional and athletic performance, especially during a hypocaloric diet [1, 2].

Insufficient dietary protein intake post-exercise may cause increased protein catabolism, which may result in a negative protein balance and slower muscle recovery [5]. This may lead to muscle wasting (e.g. sarcopenia) and training intolerance [5]. However, dietary protein intake among recreational athletes and adults engaging in intentional weight loss via caloric restriction is often insufficient to avoid muscle wasting [1, 5]. Other susceptible populations include aesthetic athletes, such as dancers, gymnasts, and bodybuilders, and athletes who must meet weight requirement, such as boxers and wrestlers [5]. There is evidence suggesting that preserving muscle mass requires ingesting a sufficient amount of high quality protein [1, 10].

Many athletes and fitness participants consume protein or amino acid supplements to maintain essential amino acid availability and stimulate lean tissue preservation. The combination of high quality protein and resistance exercise is suggested to have a synergistic effect on muscle mass preservation during intentional weight loss [1]. Nutritional supplements such as branched-chain amino acids (BCAA; valine, leucine, isoleucine) may augment or stimulate skeletal muscle regeneration by suppressing post-exercise protein degradation, therefore leading to greater gains in lean mass [5].

BCAAs are catabolized in the muscle and have been shown to regulate skeletal muscle protein synthesis and muscle recovery [11]. BCAAs may delay fatigue and stimulate muscle protein synthesis leading to post-exercise muscle recovery, allowing consumers to train longer at a higher intensity [5, 8]. Numerous studies have reported the effectiveness of a BCAA supplementation in promoting and regulating protein synthesis and suppressing endogenous protein degradation post-exercise [5, 12, 13]. Shimomoura et al. [12] found that oral ingestion of a BCAA supplement before or after exercise improved the recovery of damaged muscles by suppressing the endogenous muscle-protein breakdown during exercise [12]. Similarly, Norton & Layman [14] found that the consumption of leucine, one of three BCAAs, can turn individuals from a negative to a positive whole body protein balance after intense resistance training exercise [14]. Thus, the use of a BCAA supplement in conjunction with a resistance exercise training regimen may enhance training adaptations in recreational and advanced athletes, and benefit those with or at risk for sarcopenia [15, 16].

However, what is not known is how trained individuals participating in regular resistance training while observing calorically restricted to purposely decrease fat mass respond to BCAA supplementation. Therefore, the purpose of this study was to determine the effectiveness of a BCAA supplement on body composition, metabolism, and muscular fitness in young adult males following a carbohydrate and caloric restricted cut diet while maintaining a vigorous resistance training protocol. A cut diet is utilized to reduce fat mass while maintaining lean muscle mass by restricting calories and carbohydrate intake.

The addition of BCAAs to an athlete’s diet may allow the athlete to train longer at a higher intensity and aid in recovery, promoting greater increases in desired outcomes (i.e. strength, endurance, power, body fat, lean mass, etc.) [13]. We hypothesize that daily BCAA supplementation in conjunction with a heavy resistance training protocol and a cut diet will maintain lean body mass and decrease fat mass in resistance-trained males.

## Methods

### Experimental protocol

For 8 weeks subjects were prescribed a carbohydrate and calorically-restricted diet individually calculated based upon pre-intervention body composition and resting metabolic rate (RMR). It was made clear to subjects that the diet prescription was to be followed for the duration of the study and they were told that no nutritional supplements, other than those supplements provided, were to be ingested. In a single-blind, matched group design, subjects were provided a body building style split resistance training program for 8 weeks (four days/week). Further, subjects were randomized to pre-exercise and post-exercise ingestion of either a BCAA nutritional supplement (Scivation XTend™, Scivation, Inc.) or a carbohydrate based supplement (POWERADE®). All assessments of muscle performance and body composition were completed prior to the initiation of the prescribed diet, first dose of supplement and initiation of resistance training program, and immediately after the conclusion of the 8 week intervention period. Pre and post testing sessions were conducted in the same order and were administered in the Human Performance Laboratory in the Silcox Center at the College of Charleston. Data from another study with similar study methodology have been published, [17] thus what follows is a truncated explanation of study procedures.

### Participants

Seventeen males (between the ages of 21 and 28) who self-reported as resistance trained (defined as consistent whole body resistance training for at least 2 years prior to the onset of the study) volunteered for the study. Exclusion criteria included: less than two (2) years of prior resistance training experience, lower or upper extremity surgery within the past year, recent musculoskeletal injury, epilepsy, or another medical condition that would be exacerbated by the consumption of protein. (i.e. excessive consumption of alcohol, diabetes, Lou Gehrig’s disease, or branched-chain keto acidura). After signing the informed consent form, subjects completed a Physical Activity Readiness Questionnaire (Par-Q) to ensure that the required health status and physical activity habits for participation in this research were met. The Institutional Review Board of the College of Charleston granted approval of all study procedures.

### Body composition assessment

Total body mass was measured on a digital medical scale (Tanita, Tokyo, Japan) and height was measured using a standard medical stadiometer (Seca, Chino, CA). Percent body fat, fat mass, and fat-free mass were determined using hydrostatic weighing.

### Muscular fitness assessment

To assess muscular strength, each subject performed a one-repetition maximum (1RM) bench press and a 1RM parallel back squat using the National Strength and Conditioning (NSCA) protocol for a 1RM. Subjects were then asked to complete as many repetitions as possible at 80 %1RM for the bench press and parallel back squat. Research assistants spotted and supervised all lifts.

### Resting metabolic rate

Resting metabolic rate (RMR) was measured (ParvoMedics TrueOne® metabolic cart) following a 45 min period in which participants laid as quiet and motionless as possible under the supervision of a research assistant who ensured the subjects remained awake. Expired air was measured with the use of a plastic canopy, thus preventing the need for a facemask or mouthpiece, which may artificially elevate resting metabolic rate. Participants were instructed to freely inhale and exhale during the 30 min test.

### Dietary analyses

**Fig. 1:**
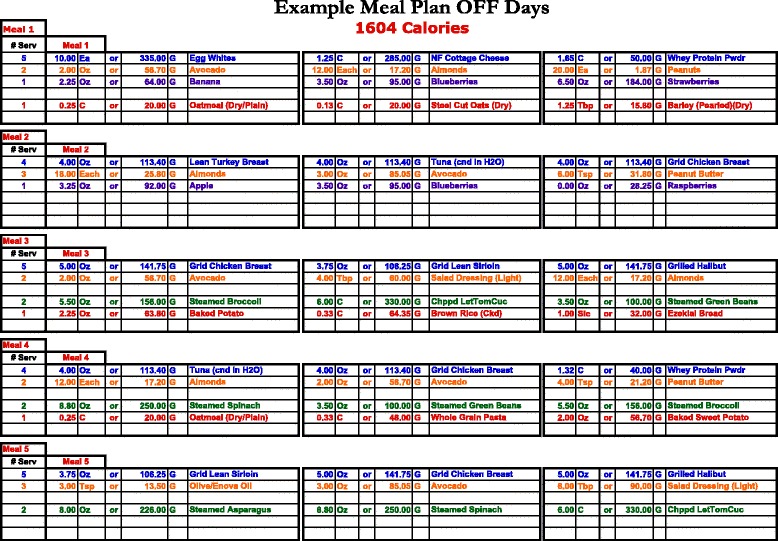
Sample dietary card for a subject during an off, non-workout, day. The Harris Benedict formula with an activity factor of 1.35 (lightly active individual engaging in light exercise 1–3 days/week) was used for workout days and 1.125 (sedentary individual) for off days

Subjects were provided an individualized caloric-restricted diet based on individual data (body mass, body composition, resting metabolic rate, etc.). All subjects, regardless of group, followed the same diet, which was designed by an industry consultant with prior experience consulting with physique athletes during pre-contest preparation. The caloric-restricted diet was designed as an 8 week “cut diet” for reducing body fat, and used a modified carbohydrate-restricted diet approach (percent of total calories for workout days were 30 % carbohydrates, 35 % protein and 35 % fat and for off days were 25 % carbohydrates, 40 % protein and 35 % fat). Each individual’s daily caloric and macronutrient intake was determined using the Harris Benedict formula with an activity factor of 1.35 (lightly active individual engaging in light exercise 1–3 days/week) for workout days and 1.125 (sedentary individual) for off days. Subjects were given a diet card (See Fig. 1) for work out days and off days that listed the total caloric goal with three meal options per meal to attain the desired intake. Mean caloric intake and macronutrient composition of the initial 4 week diet for each group are presented in Table 1. The dietary intake needs were re-calculated after 4 weeks of the study to account for any changes in body mass. Subjects were required to maintain the diet provided for them for the entire 8 week study period and weekly interviews with subjects were incorporated to help achieve compliance.

**Table 1:** Sample macronutrient breakdown during workout days and off days for  a study subject

		Caloric Intake (Kcal/day)	Protein (g)	Carbohydrate (g)	Fat (g)
Workout Day	BCAA	2456	215	184	96
	CHO	2717	238	204	106
Off Day	BCAA	2046	205	128	80
	CHO	2264	226	142	88

Each individual’s daily caloric and macronutrient intake was determined using the Harris Benedict formula with an activity factor of 1.35 (lightly active individual engaging in light exercise 1–3 days/week) for workout days and 1.125 (sedentary individual) for off days

Subjects were screened during recruitment to ensure they were properly motivated and had the required prior experience with resistance training and strictly following a set dietary plan. In addition, subjects met weekly with a research assistant to review their workout cards, adjust loads if necessary and to review their compliance with both the respective supplement and diet plan. An equal number of subjects complained about the restrictiveness of the diet as compare to subjects that reported great satisfaction with the diet. Subjects in both groups followed the same dietary plan, which provided recommendations and substitutions for each meal (See Fig. 1).

The subjects were instructed to follow the diet as closely as possible. The subjects were highly motivated to participate in what was described during recruitment as a ‘cut diet’ designed by a registered dietician who had prior experience helping professional athletes (MMA fighters, boxers, body builders) to lose body fat for a competition. There is no reason to believe that this group of homogeneous subjects experienced with resistance training and following strict diets, would eat dramatically different foods resulting in amino acid profile differences between the two groups.

The principle investigator’s prior experience utilizing food records for dietary analyses were not as accurate as desired due to subjects often under reporting foods eaten, portion sizes consumed and omission of foods the subjects felt were not allowed. The little perceived benefit of dietary analyses that confirmed compliance with dietary instructions (in addition to anthropometric outcome measures including changes in body mass, fat mass, lean body mass) did not outweigh the negative tedious aspects of completing dietary records which could have resulted in subjects withdrawing from the study or provided inaccurate data.

### Supplementation protocol

Each participant was randomly assigned to either the BCAA supplement group (BCAA; 14 g of a BCAA nutritional supplement containing seven grams of BCAA prior to and following each workout for a total of 14 g of BCAA in 28 g BCAA commercial product) or the carbohydrate nutritional supplement (CHO; 14 g of a carbohydrate based nutritional supplement (POWERADE ®) prior to and following each workout, for a total of 28 g). Thus, subjects in both treatment groups received a 112-calorie dietary supplement at each supplementing time. Neither supplement contained any fat, while the BCAA contained no carbohydrate and the CHO contained only high fructose corn syrup and no protein or amino acids. Each subject was given a 4 week supply of their supplement with specific instructions on how to mix and when to consume. Subjects returned to the lab every 4 weeks to receive additional supplement. Subjects were prohibited from consuming any other nutritional supplements during the study.

### Resistance training protocol

All subjects performed a progressive bodybuilding split style resistance-training program 4 days per week for the 8 week study duration. Subjects kept a training log during the training period and returned to the lab after 4 weeks to have their training logs reviewed. Lack of compliance with the prescript protocol was grounds for dismissal from the study.

#### Statistical analysis

To determine the effects of the BCAA supplement on body composition and muscular strength, data were analyzed (SigmaSat 3.5) using a *priori* paired and unpaired t-tests to assess changes over time and between group means, respectively. Tukey’s Test was used for post hoc analysis. Intraclass correlation coefficients (ICC) were performed to examine the test-retest reliability of the performance tests. The significance level was set at α = 0.05. Data are expressed as means ± SE.

## Results

**Fig. 2:**
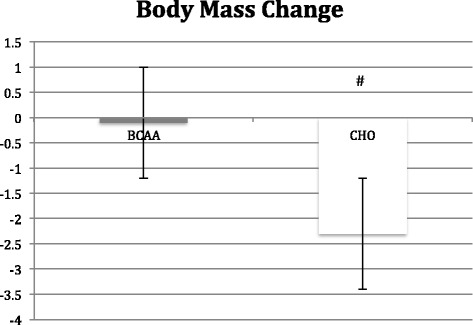
Change in body mass following 8 week study period as determined by hydrostatic weighing. BCAA group received BCAA product (14 g prior/during each workout and 14 g post workout) while the control group received 28 g carbohydrate/electrolyte mixture at the same times. All subjects followed an individualized hypocaloric diet and resistance training program. # denotes significant difference (p < 0.05) within BCAA and CHO

**Fig. 3:**
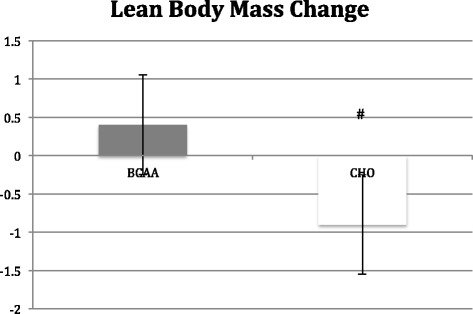
Change in lean body mass following 8 week study period as determined by hydrostatic weighing. BCAA group received BCAA product (14 g prior/during each workout and 14 g post workout) while the control group received 28 g carbohydrate/electrolyte mixture at the same times. All subjects followed an individualized hypocaloric diet and resistance training program. # denotes significant difference (p < 0.05) within BCAA and CHO

**Fig. 4:**
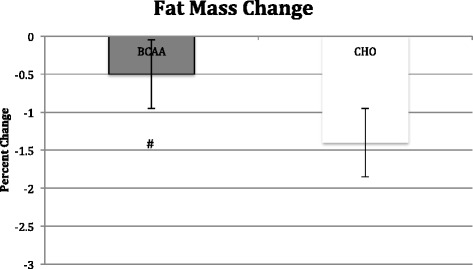
Change in fat mass following 8 week study period as determined by hydrostatic weighing. BCAA group received BCAA product (14 g prior/during each workout and 14 g post workout) while the control group received 28 g carbohydrate/electrolyte mixture at the same time. All subjects followed an individualized hypocaloric diet and resistance training program. # denotes significant difference (p < 0.05) within BCAA and CHO

Body mass did not change in the BCAA group, but the CHO group did see a significant (*p* < 0.05) reduction in body mass (−2.3 ± 0.7 kg) (see Table 2). Contributing to the change in total body mass was a significant (*p* < 0.05) loss in lean mass (−0.90 ± 0.06 kg) in the CHO group, while the BCAA group showed no change in lean mass. However, the BCAA group exhibited a significant (*p* < 0.05) decrease in fat mass (−0.05 ± 0.08 kg) that was not observed in the CHO group (See Figs. 2, 3 and 4).

**Table 2:** Changes in body mass variables before and after 8 week study period

	Age (yrs)	Height (cm)	Body Mass (kg)	Lean Mass (kg)	Fat Mass (kg)
BCAA	24.7 ± 0.6	177.9 ± 4.6	84.3 ± 5.2	72.2 ± 4.7	12.2 ± 0.7
			84.2 ± 4.8	72.6 ± 4.3	11.6 ± 0.7^a^
CHO	23.5 ± 0.6	176.6 ± 5.6	78.3 ± 2.9	67.8 ± 2.5	10.5 ± 0.5
			76.0 ± 2.4^a^	66.9 ± 2.5^a^	9.1 ± 0.7

^a^denotes significant difference (*p* < 0.05) within BCAA and CHO

All subjects were prescribed the same hypocaloric diet and exercise programs. The BCAA group received 28 g of BCAA (14 g prior/during each workout and 14 g post workout) while the CHO group received 28 g of a carbohydrate/electrolyte supplement (14 g prior/during each workout and 14 g post workout)

**Fig. 5:**
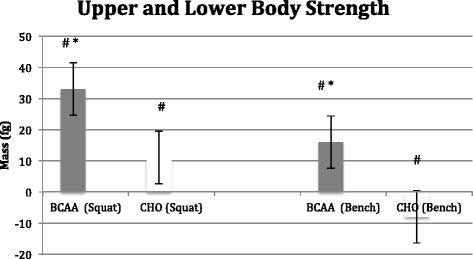
Change in muscular strength following 8 week study period as determined by 3-RM back squat and bench press. BCAA group received BCAA product (14 g prior/during each workout and 14 g post workout) while the control group received 28 g carbohydrate/electrolyte mixture at the same time. All subjects followed an individualized hypocaloric diet and resistance training program. # denotes significant difference (*p* < 0.05) within BCAA and CHO * denotes significant difference (*p* < 0.05) between BCAA and CHO

**Fig. 6:**
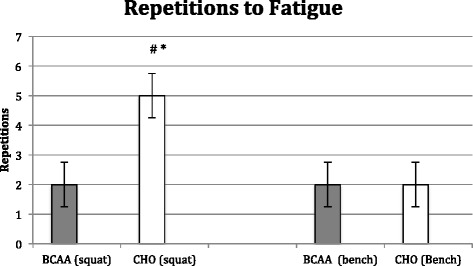
Change in muscular endurance following 8 week study period as determined by repetitions to fatigue at 80 % of estimated 1-RM on back squat and bench press. BCAA group received BCAA product (14 g prior/during each workout and 14 g post workout) while the control group received 28 g carbohydrate/electrolyte mixture at the same time. All subjects followed an individualized hypocaloric diet and resistance training program. # denotes significant difference (*p* < 0.05) within BCAA and CNO * denotes significant difference (*p* < 0.05) between BCAA and CHO

Both groups significantly (*p* < 0.05) increased lower body strength, but the change in the BCAA group (15.1 ± 2.2 kg) was significantly greater (*p* < 0.05) than the CHO group (4.8 ± 1.8 kg). The BCAA group also significantly increased upper body strength (7.1 ± 1.6 kg; p < 0.05), while the CHO group decreased strength (−3.7 ± 2.3 kg; *P* < 0.05), resulting in a significant difference between groups (*p* < 0.01). The CHO group exhibited an increase in repetitions to fatigue (5.3 ± 0.2; *p* < 0.05) on the squat exercise with no changes observed in the BCAA group. Neither group showed a significant change in repetitions to fatigue on the bench press (See Figs 5 and 6).

Finally, the BCAA group decreased RMR (−412 ± 67 kcal/day; *p* < 0.05) from pre to post observation, however this change was not different than the CHO group, who exhibited no change in RMR.

## Discussion

The purpose of this study was to examine the effects of BCAA supplementation in conjunction with resistance training and a “cut diet” on indices of muscle performance (strength and endurance) and body composition (fat mass and lean mass) in healthy resistance-trained males. We hypothesized that a pre- and post-training dose of 14 g of a BCAA supplement would improve muscle performance and decrease fat mass while maintaining lean body mass in resistance-trained males. The findings of this study support our hypothesis, as we demonstrated that 8 weeks of BCAA supplementation, resistance training, and “cut diet” had a preferential positive effect on body composition and muscular performance, compared to a group who consumed carbohydrate instead of BCAA. The observed benefits of BCAA supplementation in conjunction with resistance training and caloric restriction are important for competitive weight class athletes, the aesthetic athlete, recreationally active adults, and others who aim to lose body fat and increase or maintain lean body mass for performance and/or health reasons.

Both BCAA and CHO groups engaged in identical, supervised resistance training programs, and received individualized hypocaloric carbohydrate restricted diets for a duration of 8 weeks. Therefore, changes seen in body composition and muscle performance were likely due to the treatment (BCAA) effects rather than training effects. This is important because protein supplements such as BCAA are relied upon within a variety of populations to maintain or improve muscle mass, aid in muscle recovery, and enhance athletic performance [1, 2]. Whereas there is ample evidence for the attenuation of lean mass during a cut diet in overweight and untrained populations [2, 18, 19], there is a paucity of studies utilizing the unique combination of a resistance training program, isolated BCAA supplement, and cut diet with which to compare our study. Therefore, the results of this study have important implications for expanding understanding for developing nutrition and exercise programs for both athletes and untrained individuals.

### Body composition

Both the BCAA and CHO group exhibited changes in body composition, though the groups responded differently to the intervention. The loss in body mass in the CHO group was anticipated; as this is a typical outcome to reduced carbohydrate caloric restriction [20]. While there was a significant decrease in lean mass in the CHO group, there was not a loss of fat mass, though the trend (*p* < 0.1) was strong. This could potentially be due to the presence of high fructose corn syrup (HFCS) in the CHO supplement as HFCS, and other processed carbohydrates such as sucrose, have been associated with fat accumulation [21, 22]. However, it should be noted that the literate is not in agreement that HFCS consumption leads to fat accumulation [23]. The BCAA group showed no change in body mass, due to the maintenance of lean mass in the presence of a significant loss of fat mass. These results differ slightly from a similar study performed by Mourier et al. [24] who found that restricted calorie intake and BCAA supplementation among competitive male wrestlers exhibited a significant reduction in abdominal adipose tissue, compared to high protein, low protein, and control groups [24].

We anticipated that the BCAA group would maintain lean mass at the conclusion of the study, and as anticipated the BCAA group maintained lean body mass, compared to the CHO group who lost lean body mass. These results indicate the effectiveness of the BCAA supplement compared to the carbohydrate placebo at promoting lean mass maintenance. This finding is consistent with other studies that have reported enhanced skeletal muscle protein synthesis and lean muscle maintenance in response to exercise and BCAA supplementation [13, 25].

It is possible that the decreased lean mass in the CHO group can be attributed to a decrease in protein synthesis, due to a reduced calorie diet, coupled with a muscle-damaging resistance training program. It has been shown that BCAA supplementation enhances/promotes myofibrillar protein synthesis and aids in muscle recovery. Some researchers have found that BCAA supplementation post-exercise attenuated the decline in myofibrillar protein synthesis, which is vital in preserving lean mass during weight loss [8, 26]. Thus, the addition of BCAA supplements may have allowed for the maintenance of lean muscle mass because of its potential to enhance lean muscle protein synthesis.

Some studies have indicated a possible dose–response relationship regarding BCAA supplementation and body composition, including a study conducted by Spillane, Emerson, and Willoughby [27]. Subjects were provided with 9 g/day of a BCAA supplement combined with 8 weeks of heavy resistance training, and found no preferential effect of BCAA supplementation on body composition [27]. However, Mourier et al. [24] found that a high daily dose of a BCAA supplement reduced body fat and spared lean mass in male athletes [22]. Similarly, the 26 g daily ingestion of BCAA product in our study had a beneficial effect of reduced fat mass and lean mass maintenance.

The preservation of lean mass is important for both athletic populations striving to improve athletic performance, and for older or sedentary populations at risk for obesity-related or age-onset obesity or sarcopenia and other age-related diseases [4, 6, 16]. Providing individuals with BCAA can stimulate myofibrillar protein synthesis and in turn preserve lean body mass. Our data suggests that BCAA supplementation may be effective in individuals attempting to lose fat mass while maintaining lean mass.

There is a paucity of studies investigating the use of BCAAs with a hypocaloric diet and resistance training, but some studies attempt to elucidate the connection between some aforementioned factors. In a study investigating a hypocaloric diet in conjunction with increased dietary protein intake, Mettler et al. [25] found that providing a higher percentage of daily caloric intake by protein (35 %) was more effective in maintaining lean body mass during a hypoenergetic diet than 15 % dietary protein intake [25]. However, these researchers sourced protein intake from dietary foods, rather than a supplement such as BCAA.

Coker et al. [2] found that essential amino acids supplementation was effective for preserving lean muscle mass even without exercise [2]. Researchers illustrated that the combination of a protein supplement (whey and essential amino acids) with a calorie restricted diet was more effective than a hypocaloric meal replacement control at concurrently reducing adipose tissue and preserving lean tissue during the caloric restriction-induced weight loss in elderly obese subjects [2]. Similarly, BCAA supplementation was found to provide a beneficial effect on body composition and isometric hand-grip strength, even without a concurrent exercise training protocol [28]. Researchers demonstrated that 30 days of ingesting 14 g of a BCAA supplement significantly lean mass and hand-grip strength in untrained males. However, no control group was provided for this study, making the conclusions as to whether the BCAA supplementation was predominant in improving lean mass and grip strength inconclusive [28].

### Metabolism

Eight weeks of resistance training combined with a BCAA supplement and caloric restriction elicited a significant difference in RMR between BCAA and CHO groups, where the BCAA group decreased RMR and the CHO group showed no changes. The amount of lean tissue mass is essential in determining metabolic rate, where a greater amount of lean tissue increases RMR. Lean tissue is more metabolically active than fat tissue, and requires more energy at rest; thus increased energy expenditure can then decrease risk for chronic diseases such as metabolic disease, diabetes mellitus, and cardiovascular disease [6]. Therefore, the insignificant increase in lean mass in the BCAA group would not contribute to a significant change in RMR.

### Muscle strength and endurance

Resistance training, provision of adequate amounts of dietary protein, and essential amino acids have all been shown to increase muscle protein synthesis in healthy adults [8], and this is essential to maintaining muscle fitness In this study, the BCAA supplement maintained lean mass while significantly improved participants’ 1RM squat and 1RM bench press from pre-test and was more effective than the CHO group, indicating that BCAA supplementation was effective in developing muscular strength in trained subjects during caloric restriction. Similarly, Tsujimoto et al. [29] investigated the effects of BCAA on training volume following 5 weeks of resistance training and daily ingestion of BCAA and found that BCAA supplementation increased maximal strength in bench press and squat exercises [29].

However, a recent study by Spillane et al. [27] utilized a reduced daily dosage of BCAA supplement (9 g/day; 4.5 g pre- and post-exercise, compared to our 28 g per training day) combined with an 8 week resistance-training program [27]. Muscle strength increased with training, but no significant effects were evident between placebo and BCAA groups, indicating the lack of a treatment effect in the BCAA group. The results of this study suggest that there may be a dose–response relationship influencing the effectiveness of a BCAA supplement, where a greater dosage of BCAA induces greater performance benefits [29]. It is unlikely that the training duration of this study influenced results, as Tsujimoto et al. [29] found significant performance benefits with only 5 weeks of resistance training. Further, it may be that in a hypocaloric state the BCAA has a more robust effect [30].

It is difficult to explain the increase in repetitions to fatigue on the parallel squat in the CHO group, with no other changes being observed within or across groups. It was anticipated that on a hypocaloric diet, there would not be any gains in muscular endurance, given the glycolytic nature of the activity [30, 31]. It is possible that the CHO supplementation in the CHO group enhanced glycogen storage, which improved fatigue resistance and in turn resulted in increased repetitions to fatigue. However, our small sample size allows for robust changes in a few subjects to result in significant group improvements. We observed consistent and nearly uniform responses from subjects across the other measures of muscular performance, but in the repetitions to fatigue a couple subjects showed tremendous improvements, thus resulting in a significant group change. Additionally, with human performance testing it is possible these subjects did not perform to their maximal ability during pre-testing data collection for a variety of reasons (fatigue, distraction, lack of effort, etc.).

Though there remains controversy regarding the effectiveness of BCAA supplementation on muscle performance and body composition among both trained and untrained persons, there is a greater amount of consensus regarding the effects of BCAA supplements on muscle damage and recovery, which may in turn inform muscle performance (strength and endurance). Shimomoura et al. [12] found that oral ingestion of a BCAA supplement before or after exercise improved the recovery of damaged muscles by suppressing the endogenous muscle-protein breakdown during exercise (decreasing the release of essential amino acids from exercising muscles) [12]. This in turn may have implications in improved muscle performance and recovery. However Ra et al. [26] found that BCAA supplementation alone was not sufficient to inhibit muscle soreness and damage after a damaging bout of eccentric exercise [26].

## Conclusions

The variability in experimental approaches adopted by researchers, and the factors investigated, such as the supplement quantity, treatment duration, timing of ingestion, training status and intensity, and dietary control, make direct comparisons of studies difficult. It is thus difficult to conclusively quantify the benefits of BCAA supplements across populations. However, our data suggest that under hypocaloric conditions, those who participate in heavy resistance training can maintain lean mass and muscular performance by utilizing a BCAA product pre and post workout. Further, while this protocol resulted in a loss of lean mass in the CHO group, the improvement in lower body strength and repetitions to fatigue suggest that minimal CHO supplementation on a cut diet may help to maintain some performance measures.
